# A Large Intergenic Spacer Leads to the Increase in Genome Size and Sequential Gene Movement around IR/SC Boundaries in the Chloroplast Genome of *Adiantum malesianum* (Pteridaceae)

**DOI:** 10.3390/ijms232415616

**Published:** 2022-12-09

**Authors:** Xiaolin Gu, Ming Zhu, Yingjuan Su, Ting Wang

**Affiliations:** 1 College of Life Sciences, South China Agricultural University, Guangzhou 510642, China; 2 School of Life Sciences, Sun Yat-sen University, Guangzhou 510275, China; 3 Research Institute of Sun Yat-sen University in Shenzhen, Shenzhen 518057, China

**Keywords:** *Adiantum malesianum*, genome comparison, inverted repeat, chloroplast conformations, topological tension

## Abstract

Expansion and contraction (ebb and flow events) of inverted repeat (IR) boundaries occur and are generally considered to be major factors affecting chloroplast (cp) genome size changes. Nonetheless, the *Adiantum malesianum* cp genome does not seem to follow this pattern. We sequenced, assembled and corrected the *A. flabellulatum* and *A. malesianum* cp genomes using the Illumina NovaSeq6000 platform, and we performed a comparative genome analysis of six *Adiantum* species. The results revealed differences in the IR/SC boundaries of *A. malesianum* caused by a 6876 bp long *rpoB-trnD-GUC* intergenic spacer (IGS) in the LSC. This IGS may create topological tension towards the LSC/IRb boundary in the cp genome, resulting in a sequential movement of the LSC genes. Consequently, this leads to changes of the IR/SC boundaries and may even destroy the integrity of *trnT-UGU*, which is located in IRs. This study provides evidence showing that it is the large *rpoB-trnD-GUC* IGS that leads to *A. malesianum* cp genome size change, rather than ebb and flow events. Then, the study provides a model to explain how the *rpoB-trnD-GUC* IGS in LSC affects *A. malesianum* IR/SC boundaries. Moreover, this study also provides useful data for dissecting the evolution of cp genomes of *Adiantum*. In future research, we can expand the sample to Pteridaceae to test whether this phenomenon is universal in Pteridaceae.

## 1. Introduction

Chloroplasts (cp) are important sites for plant photosynthesis and play an important role in the biosynthesis of carbohydrates, proteins and lipids in plants [1]. They consist of a double-stranded genome with a genetic system that is independent of the nuclear genome [1,2]. Typical cp genomes of terrestrial plants are 120–160 kb in size, and their structures are mostly quadripartite [3]. They usually contain about 120 genes, including protein-coding genes, ribosomal RNAs (rRNAs) and transfer RNAs (tRNAs) [4,5]. Cp genome structure is generally conserved in terms of gene structure, gene content and gene order [6,7]. However, cp genomes also exhibit some structural variation, such as uneven expansion and contraction of inverted repeat (IR) [7], which is a dynamic process where genes are transferred from single-copy (SC) to IRs or sequences are inserted into IRs, leading to IR expansion [8]; inversely, if genes are transferred from IRs to SC, or if sequences are deleted in IRs, this will cause IR contraction [9]. Even within closely related taxa, the changes in IR border genes can vary, a process referred to as “ebb and flow” [9,10], and this process is often linked to genome size change [11,12]. For example, the size of the IRs in angiosperms is generally between 20 and 30 kb [13], while in most non-seed plants, the size only ranges from 10 to 15 kb [14]. However, some extreme cases exist, such as *Pelargonium* x *hortorum* (~76 kb) [15], *Monsonia speciosa* (7 kb) [16], or the loss of IRs of Pinaceae and some other species [17,18,19,20]. If the expansion of IRs is caused by genes shift between IRs and SC, it will be accompanied by a decrease in SC. For instance, small single-copy (SSC) of *Lamprocapnos spectabilis* and some Ericaceae species are quite short and only contain part of a gene or one gene [21,22,23,24]. All the SSC of *Asarum canadense* and *A. sieboldii* var. *sieboldii* are incorporated into IRs [25].

Generally, it is believed that the contraction of IRs is related to the deletion/loss of DNA in one IR copy [7,20,21]. However, different explanations exist for the expansion of IRs. Goulding et al. proposed two mechanisms to explain IR expansion. One is the gene conversion mechanism, which assumes that recombination between two copies of IR occurs continuously, forming a heteroduplex by a Holliday junction; then, expansion of IRs is caused by heteroduplex DNA repair [9]. However, gene conversion can only account for small-scale expansion. As for mass expansion, it is thought to occur through double-strand break events (DSBs). Namely, a double-strand DNA break occurring in one IR copy will be repaired through a repair mechanism, which may extensively incorporate SC sequences into IRs, resulting in IR expansion [9,26]. In addition, some studies suggest that the change in IR size is due to homologous recombination between repeated sequences. For example, the unusually large IRs of geranium (*Pelargonium hortorum*) are thought to be generated via multiple inversions mediated by repeated sequences near their endpoints; i.e., these inversions may be caused by recombination between homologous dispersed repeats [11]. During the diversification of complex thalloid liverworts, the amplification of its IRs is believed to be the result of homologous recombination caused by the increase of SSR near the IR/LSC boundary [27]. The loss of IRs in cupressophyte and Pinaceae is also suggested to be associated with homologous recombination between short repeats [17,28]. Likewise, the amplification of *Lamprocapnos spectabilis* IRs is considered to be related to the inversion of IRs, which may be caused by repeats [23].

In contrast to the previously proposed mechanisms, this study notes that large intergenic spacers (IGSs) are the main factor governing cp genome size change in *Adiantum malesianum*. We conducted a genome-wide comparison for six *Adiantum* cp genomes, including a comparative analysis of IR/SC boundaries and repeats. A very large *rpoB-trnD-GUC* IGS (6876 bp) was identified in the large single-copy (LSC) in *A. malesianum*, while the length of its corresponding components is <1 kb in other species. Along with causing the increase in *A. malesianum* cp genome size, we have also detected that this large *rpoB-trnD-GUC* IGS has effects on gene location and integrity at IR/SC boundaries.

## 2. Results

### 2.1. Genome Assembly and Annotation

**Figure 1:**
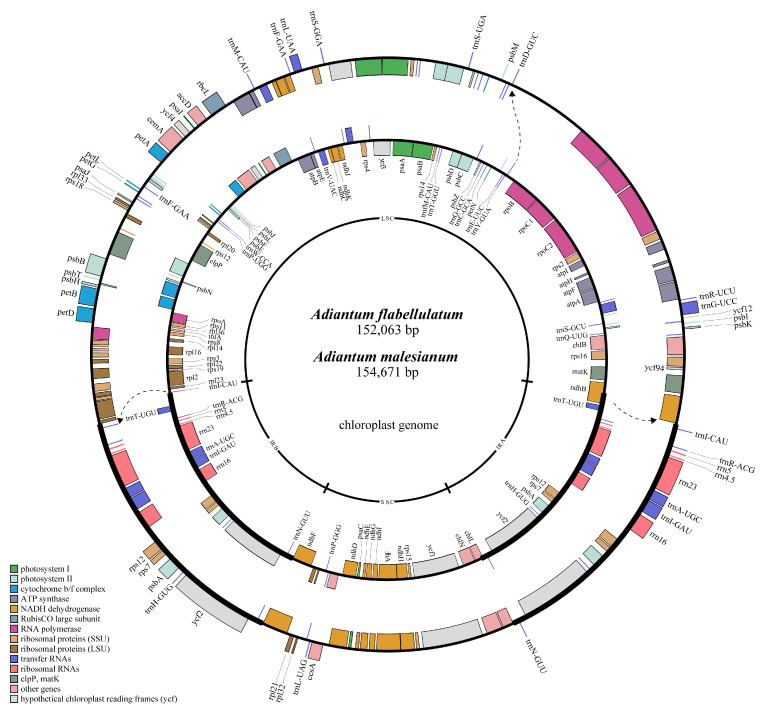
Gene map of *A. flabellulatum* and *A. malesianum* cp genomes. The inner circle is *A. flabellulatum*, while the outer is *A. malesianum*. Genes on the inside of the circle are transcribed clockwise, while those on the outside are transcribed counter-clockwise. Genes are color-coded based on functions. The dotted arrows indicate the position shift of the same gene in two cp genomes. The thicker black line represents the IRs. Names of the same genes in the two cp genomes are displayed only once.

The complete cp genomes of *A. flabellulatum* and *A. malesianum* are 152,063 bp and 154,671 bp in length, respectively. They are divided into an LSC (83,384 bp, 89,030 bp) and an SSC (21,449 bp, 21,487 bp) by a pair of IRs (23,615 bp, 22,077 bp), exhibiting a typical quadripartite structure (Figure 1). Their overall GC content is 43.3% and 42.6%, respectively, among which IRs make up the greatest percentage (46.7%, 46.6%), followed by LSC (42.5%, 41.8%) and SSC (39.2%, 37.7%). The two complete cp genomes contain 130 and 131 genes, respectively. Compared with *A. flabellulatum*, *A. malesianum* has two fewer copies of *trnT-UGU*, and it has one more copy of *trnI-CAU*, *trnF-GAA* and *ycf94*. Notably, the *rps12* is a trans-spliced gene, in which two 3′ end exons are repeated in IRs and a 5′ end exon is situated in LSC. The detailed genome components are shown in Table S1.

### 2.2. Chloroplast Genome Comparison among Adiantum

The cp genome size of the six *Adiantum* species ranges from 149,956 bp to 154,671 bp (Table 1). The length of LSC of the five species is about 82–83 kb, but the length of *A. malesianum* is up to 89 kb larger than others. SSC and IRs are slightly different in length (21,390–21,539 bp and 22,077–23,615 bp, respectively). The total GC content of the six species ranges from 42.0 to 44.3%, of which IRs (46.3–46.9%) have the highest GC content, followed by LSC (40.8–43.7%) and SSC (37.1–41.6%).

### 2.3. Inverted Repeat/Single-Copy Boundary Analysis of Adiantum

**Figure 2:**
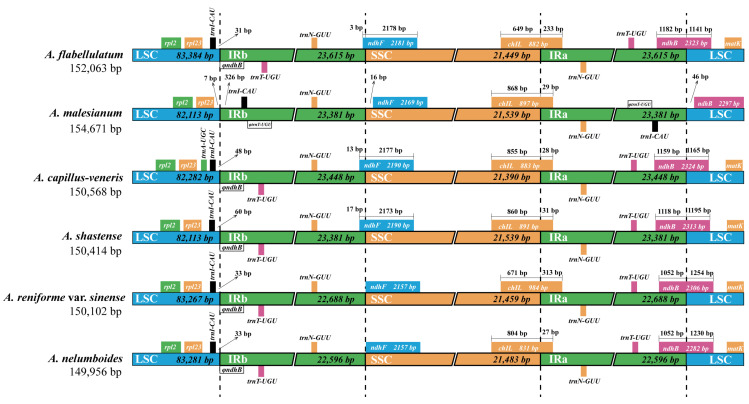
Analysis of the expansion/contraction of IR/SC boundaries in six *Adiantum* cp genomes. For each species, genes transcribed on the positive strand are depicted from right to left above the corresponding tracks, while genes on the negative strand are depicted from left to right below the tracks. Arrows indicate the distance from one side of the gene to the LSC/IR boundary. *ΦndhB* is a partial copy of *ndhB* in the IRa. *ΦtrnT-UGU* represents fragmented sequences of *trnT-UGU*.

IR/SC boundaries were recalibrated for the six species. For all the six species, genes located near the IR/LSC and IR/SSC boundaries included *matk*, *ndhB*, *trnN-GUU*, *chlL*, *ndhF*, *rpl2*, *rpl23* and *trnI-CAU*. Apart from this consistence, there exist some gene distribution differences at the boundaries between the six species, mainly caused by *A. malesianum* (Figure 2). For instance, *ndhB* genes span the IRa/LSC boundary across all species except for *A. malesianum*. All five species IRb contain *trnT-UGU* except for *A. malesianum*. The *trnI-CAU* gene is located in IRb in *A. malesianum*, but it is located in LSC, close to LSC/IRb boundary, with a distance to the boundary between 31–60 bp in the other five species. Moreover, although *chlL* genes cross the SSC/IRa boundary in the six cp genomes, the length located in IRa is significantly longer in *A. reniforme* var. *sinense* (313 bp) and *A. flabellulatum* (233 bp) than in the other four species (27–31 bp).

Expansion and contraction of IR/SC boundaries are generally considered to be the major factors inducing cp genome size change. This seems not to be the case in *A. malesianum*. Cp genome comparison of the six species (Table 1) revealed that the size change of *A. malesianum* cp genome is caused by LSC rather than IRs, because *A. malesianum* has the shortest IRs, but the largest cp genome. Importantly, IRs of *A. malesianum* had two copies of *trnI-CAU* (74 bp), but were missing part of the *ndhB* gene (~1 kb), showing a net loss of IR sequences.

### 2.4. Description of rpoB-trnD-GUC Intergenic Spacer

**Figure 3:**
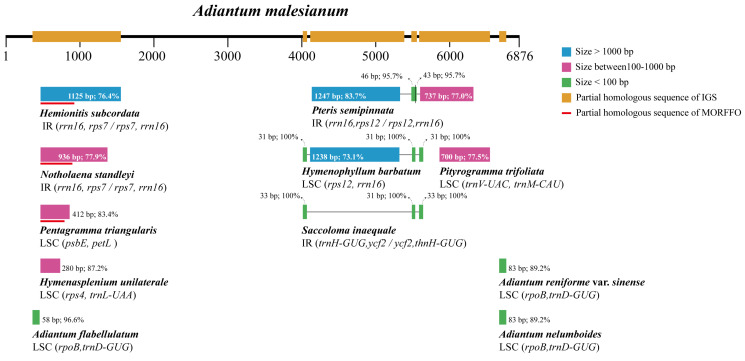
The schematic diagram of the location of homologous sequences included in the large *rpoB*-*trnD*-*GUC* IGS of *A. malesianum*. Gray lines connect different homologous sequences of one specie.

The IGS of *rpoB-trnD-GUC* in the cp genome of *A. malesianum* (6876 bp) was found to be significantly longer than that of other *Adiantum* species (669–945 bp), leading to the increase of the *A. malesianum* cp genome size. Robison et al. once proposed the concept of the Mobile Open Reading Frames in Fern Organelles (MORFFO), which refers to a group of movable insertion sequences that widely exist in fern organelles. They are usually considered to be related to structural changes of the cp genome, and may be the main driving force of cp genome structural evolution [29]. The *rpoB-trnD-GUC* IGS position of *A. malesianum* is the position where MORFFO tends to be inserted. Thus, to verify whether the insertion detected in the large IGS of *A. malesianum* cp genome is consistent with the MORFFO, MORFFO sequences were determined by local BLAST searches using the database established from *morffo1*, *morffo2* and *morffo3*, with consensus sequences of the large IGS as queries. In addition, to examine whether the large IGS identified in this study possesses mobile properties, these sequences were subjected to local BLAST searches. The results show that the large IGS in this study does not show homology with *morffo* motifs. However, we conducted a BLAST search for this IGS in GenBank and identified 11 Polypodiidae ferns dominated by Pteridaceae with homologous sequences. The homologous sequences range in size from 31 to 1247 bp with identity 73.1–100%. In other ferns, partial homologous sequences occur in other intergenic spacers rather than in the *rpoB-trnD-GUC* IGS. Noteworthily, some homologous sequences contain a *motif1* fragment, which belongs to MORFFO (Figure 3). However, we did not detect any MORFFO homologous sequence fragments in *rpoB-trnD-GUC* IGS of *A. malesianum* cp genome. This indicates that these moving inserted fragments may also be in constant change during the evolution of genome structure.

**Figure 4:**
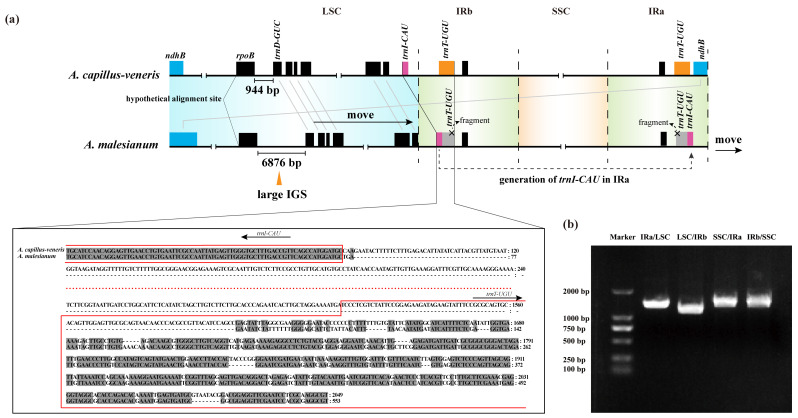
(**a**) A model to explain the structural change around IR/SC boundaries in the *A. malesianum* cp genome. It shows that the large *rpoB-trnD-GUC* IGS causes sequential gene movement in LSC, leading to *trnI-CAU* being “squeezed” into IRb and replacing part of *trnT-UGU*; then, the generation of *trnI-CAU* in IRa leads to the movement of *ndhB* from IRa into LSC. *Adiantum capillus-veneris* is used as the reference, because it has the highest level of homology with *A. malesianum*. Gene *ropB* is set as the start site of alignment. Black horizontal lines represent gene sequences; “||” represents sequence omission. Black boxes indicate identical genes; colored boxes are genes with location changes at the IR/SC boundaries. Of them, the *trnT-UGU* gene in *A. malesianum* is experimentally confirmed as unable to be normally expressed, as it only has a partial fragment remaining. Inset is the sequence alignment of *trnT-UGU*, *trnI-CAU* and their intergenic spacer between the two species; gray color indicates an identical site, red dots represent omitted bases present in *A. capillus-veneris* and “-” represents the gap. The number on the right side of the sequence is the number of bases. (**b**) Electropherogram showing the amplified DNA fragments corresponding to the four IR/SC boundaries in the *A. malesianum* cp genome.

Interestingly, this *rpoB-trnD-GUC* IGS change also causes sequential movement of LSC genes (Figure 1 and Figure 4a). The movement made *trnI-CAU* “squeezed” into IRb but without expanding IR length, because *trnI-CAU* replaced part of *trnT-UGU*. Moreover, the occurrence of *trnI-CAU* in IRa also imposed effects on *ndhB*, moving it completely out of IRa (Figure 4a). We say this because if the normal IR/SC boundaries are expanded, the *trnI-CAU* is included in the IRs, leading to the expansion of IR length without changing the integrity of its own IR/SC boundary genes. However, in the cp genome of *A. malesianum*, we accidentally matched the *trnT-UGU* fragment. This shows that the *trnT-UGU* is neither displaced nor lost, but has obviously been destroyed. Because the position of the *trnT-UGU* fragment coincides with and is closely adjacent to that of the *trnI-CAU*, it seems that the *trnI-CAU* “occupies” the position of the *trnT-UGU*.

To confirm the assembly of IR/SC boundaries in the *A. malesianum* cp genome, we designed primers corresponding to the four boundaries (IRa/LSC, LSC/IRb, IRb/SSC and SSC/IRa), conducted PCR amplification and resequenced the amplified fragments (Table 2, Figure 4b). The accuracy of the assembly was verified. Moreover, the disruption of *trnT*-*UGU* integrity was also experimentally verified by resequencing.

### 2.5. Characterization of Repeated Sequences in the Six Adiantum Chloroplast Genomes

**Figure 5:**
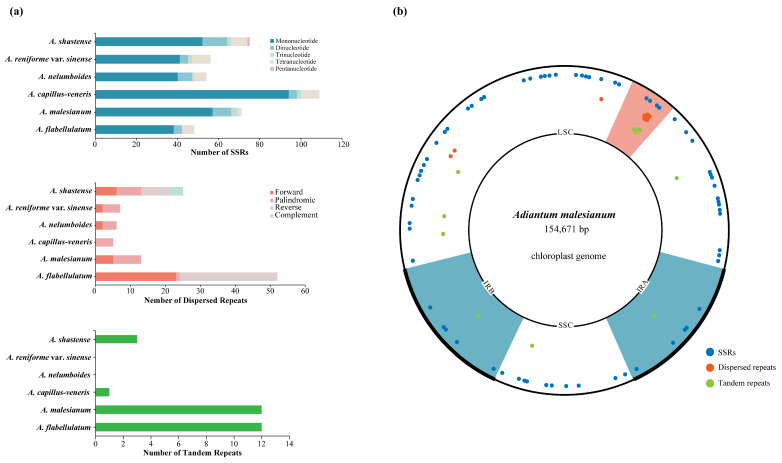
(**a**) Comparative analysis of repeated sequences in the six *Adiantum* cp genomes. The number of SSRs (**above**), dispersed repeats (**middle**) and tandem repeats (**below**) are presented. (**b**) Distribution pattern of SSRs, dispersed repeats and tandem repeats in the *A. malesianum* cp genome. Blue regions are IRs; the red region is the *rpoB-trnD-GUC* IGS. Dots indicate relative positions of the repeats.

The distribution of simple sequence repeats (SSRs) in the six *Adiantum* cp genomes was examined. As shown in Figure 5a, most were mononucleotide repeats, among which A/T motifs account for the most (42.67–65.14%). No SSRs with hexanucleotide motifs were detected. Most SSRs were located in LSC (54.55–69.01%), followed by IRs (19.72–29.36%) and SSC (8.26–20.00%). Moreover, most SSRs were located in IGS (64.79–87.50%) and introns (8.33–26.76%), while a small number (2.67–10.91%) were located in coding regions (Table S2).

Dispersed repeats over 30 bp were also detected. They differed in number and position across species (Figure 5a), with numbers varying from 53 (*A. flabellulatum* contains) to 5 (*A. capillus-veneris*) and length varying from 30 bp (*A. shastense*, *A. capillus-veneris*, *A. malesianum*, *A. flabellulatum*) to 123 bp (*A. malesianum*). Most of these repeats were distributed in IGS, followed by introns, whereas there were few in coding regions (Table S3).

Then, the tandem repeats were identified. Results with a minimum identity of 90% and a unit length of 15 bp are presented in Table S4. Except for *A. nelumboides* and *A. reniforme* var. *sinense*, the other four species possess tandem repeats (1–12), with motif sizes from 15 to 102 bp. *A. flabellulatum* and *A. malesianum* have the most tandem repeats, while *A. capillus-veneris* has the least. The proportion of tandem repeats distributed in IGS is significantly higher than that in coding regions (Figure 5a).

To further explore whether the large *rpoB-trnD-GUC* IGS of *A. malesianum* is related to repeat occurrence, the distribution of repeats in the *A. malesianum* cp genome was plotted (Figure 5b). This shows that dispersed repeats are mostly restricted to the *rpoB-trnD-GUC* IGS and concentrated within a segment of 300 bp.

## 3. Discussion

Although cp genomes are generally thought to be evolutionarily conserved [30,31], increasing evidence has shown that their structure, size and evolutionary rates can be quite variable in certain lineages, including ferns. For instance, large insertions have been detected in the IRs of Polypodiales species *Woodwardia unigemmata* and *Lepisorus clathratus*, whose sequences show high similarity to LSC fragments of Ophioglossales and Cyatheales and to the *tRNA-CGA*-*tRNA-TTT* spacer of *Asplenium nidus* mitochondrial genome [32]. To characterize factors affecting cp genome stability, Robison et al. have identified MORFFO in cp genomes of ferns and other plant groups, pointing out that they may function as mobile elements to drive cp genome structural changes, including inversions and intergenic expansions [29]. More recently, MORFFO was also found in *Hymenophyllum* and Polypodiaceae species [33,34]. In this study, the large IGS is MORFFO’s preferred position of insertion, but does not show homology with *morffo* motifs. However, partial homologous sequences of this IGS have been detected in 11 Polypodiidae ferns dominated by Pteridaceae (Figure 3), indicating that it could have multiple and complex origins. More importantly, it contains a sequence fragment homologous to MORFFO element *motif1* identified in *Pentagramma triangularis*, *Notholaena standleyi* and *Hemionitis subcordata*. This may be because *morffo* motifs are conservative regions of insertions in many fern species [29], but they actually may have diversified or changed in different species.

Expansion and contraction of IRs are frequently invoked to explain the size change of cp genomes [35,36]. Apart from this, other reasons have been increasingly put forward as well. Liu et al. reported that the change of cp genome size in Polypodiaceae is mainly caused by several large insertions occurring in the intergenic spacer region [33]. For *Cypripedium* (Orchidaceae), Guo et al. pointed out that its cp genome expansion is strongly correlated with the proliferation of AT-biased non-coding regions [37]. As for *Inga* (Leguminosae) and *Acacia* (Leguminosae), the increase of their cp genome size is attributed to the combination of tandem repeat expansion and IR expansion [38]. In this study, the differences in IR/SC boundaries between the six *Adiantum* cp genomes are mainly caused by *A. malesianum*, but these changes in the IR/SC boundaries are not enough to explain the significant differences in its genome size. For example, in the largest cp genome *A. malesianum*, although *trnI-CAU* enters the IRs and adds two copies of *trnI-CAU*, more bases are removed from the IR (*ndhB*). Thus, the increase caused by the large *rpoB-trnD-GUC* IGS (6876 bp) is more reasonable. In addition, to detect changes in the IR/SC boundaries and whether large IGS is related to repeats, we also gathered statistics on the number and distribution of repeats of six *Adiantum* cp genome. In *A. malesianum*, SSRs are relatively evenly distributed throughout the cp genome, while dispersed repeats are mainly limited to *rpoB-trnD-GUC* IGS and concentrated within a segment of 300 bp. No specific repeats were found at the IR/SC boundaries of *A. malesianum*, indicating that the change of IR/SC boundaries in this study was not caused by homologous recombination of repeats.

Previous studies have shown that the expansion or contraction of IRs usually affects the conformation of its cp genome; for example, IR expansion usually decreases with SC shortening [7,23,25,39]. Nonetheless, in this study, we noticed that the entrance of *trnI*-*CAU* into IRs does not cause the increase of IR size in *A. malesianum*, as it replaces a partial sequence of *trnT-UGU*. We speculate that this is due to the asymmetric and directional influence of the size increase of the *rpoB-trnD-GUC* IGS. Compared with other cp genomes, *ndhB* of the other five species crosses the LSC/IRa boundary. However, the *ndhB* of *A. malesianum* is completely separated from IRa. This indicates that *ndhB* in *A. malesianum* may move towards LSC. If this asymmetric topological tension is towards IRa, it conflicts with the phenomenon of *ndhB* moving towards LSC. On the contrary, this asymmetric topological tension is towards IRb, because *trnI-CAU* has been transferred to IRb; more importantly, other LSC genes are also sequential moving toward IRb (Figure 1 and Figure 4a). As a result, this large IGS in *A. malesianum* not only increases the cp genome size, but also drives the sequential movement of genes in LSC, “squeezing” *trnI-CAU* and shifting it from LSC into IRb. This movement ends with the destruction of *trnT-UGU*, because the position and order of genes at the IRb-SSC-IRa boundaries and/or inside of the six cp genomes are similar. Differently, the displacement of *ndhB* is further driven by the generation of *trnI-CAU* in IRa, moving *ndhB* completely out of IRa into LSC. Thus, this also provides favorable evidence that the dynamic change of the IR/SC boundaries occurs first in IRb.

## 4. Materials and Methods

### 4.1. Sample Collection, DNA Extraction and Sequencing

Fresh leaves of *A. flabellulatum* and *A. malesianum* were sampled from the campus of South China Agricultural University (SCAU), location (E113°35′, N23°16′), altitude: 35 m, quickly frozen in liquid nitrogen and stored in an ultra-low-temperature refrigerator at −80 °C until use. The specimens were stored in the Herbarium of the College of Life Sciences, SCAU (specimen no.: GXL20210901, GXL20210902).

The Tiangen Plant Genome DNA Kit (Tiangen Biotech Co., Ltd., Beijing, China) was utilized to extract DNA according to the manufacturer’s instructions. DNA quality was inspected on 1% agarose gels and DNA concentration was measured by DNA Assay Kit in Qubit 3.0 Fluorometer (Invitrogen, Life Technologies, Carlsbad, CA, USA). After quality assessment, the library was constructed by sonicating the genomic DNA sample to 350 bp. Then, DNA fragments were end-polished, A-tailed and ligated with the full-length adapter for Illumina sequencing, followed by further PCR amplification. The sequencing library was generated using NEBNext Ultra DNA Library Prep Kit for Illumina (NEB, Ipswich, MA, USA) following the manufacturer’s recommendations. The Illumina NovaSeq6000 platform was used for sequencing.

The positioning of the four boundaries in the cp genome was experimentally confirmed by primers, PCR and re-sequencing. Primers were designed using SnapGene V6.1 (https://www.snapgene.com, accessed on 28 June 2022). The TIANMO BIOTECH DNA Assay kit (Beijing, China) was used according to the manufacturer’s protocol to extract total DNA from the sample. A thermal cycler (T100, Bio-Rad, Hercules, CA, USA) was used for PCR with the following conditions: 95 °C for 5 min, 34 cycles of 95 °C for 30 s, 54 °C for 30 s and 72 °C for 1 min 30 s. PCR products were analyzed by electrophoresis (1% agarose in 1× TAE buffer) and then sequenced using the Sanger ABI 3730 method.

### 4.2. Genome Assembly and Annotation

Raw reads were recorded in FASTQ format. Low-quality reads were filtered out by Fastp v0.19.7 [40]. The clean reads were used to assemble the cp genome by GetOrganelle [41] with the complete cp genome of *A. capillus-veneris* (NC_004766) as the reference and NUMER [42] was used to check their collinearity. BWA [43] was used to map raw data to the assembled cp genome (Table S5). The cp genome was annotated with GeSeq [44] with *A. capillus-veneris* (NC_004766) as the reference and manually corrected based on comparison with *A. nelumboides* (NC_050350) with Geneious Prime [45]. The final cp genome circle map was completed using OGDraw [46]. The GenBank accession numbers obtained after uploading to the NCBI (National Center for Biotechnology Information) are NC_064144.1 and NC_063331.1, respectively.

### 4.3. Comparative Genomic Analysis of Adiantum Chloroplasts

*A. flabellulatum*, *A. malesianum*, *A. capillus-veneris*, *A. nelumboides*, *A. reniforme* var. *sinense* (NC_062433) and *A. shastense* (NC_037478) were used for the whole-cp genome comparison, including genome size, gene content, IR/SC boundaries and base composition.

### 4.4. Statistical Analysis of Repeated Sequences

SSR loci were analyzed using MISA [47]. The numbers of iterations for mono-, di-, tri-, tetra-, penta- and hexanucleotide repeat motifs were 10, 5, 4, 3, 3 and 3, respectively.

Finder v4.09 [48] was used to identify tandem repeats in the six *Adiantum* cp genomes, with match, mismatch and indel set as 2, 7 and 7, respectively. The minimum alignment score and the maximum period were set to 90 and 500, respectively.

The position and size of dispersed repeats (forward, reverse, complement and palindromic) in cp genome sequences were detected using REPuter online software [49] (https://bibiserv.cebitec.uni-bielefeld.de/reputer, accessed on 1 May 2022), with a repeat size of ≥30 bp and a Hamming distance of 3.

## 5. Conclusions

We assembled and annotated the first complete cp genome of *A. flabellulatum* and *A. malesianum* and performed a genomic comparison of six *Adiantum* species. We identified a 6876 bp long IGS between *rpoB* and *trnD-GUC* in the *A. malesianum* cp genome, which contains homologous sequences from 11 Polypodiidae species and concentrated dispersed repeats. The large size of *rpoB-trnD-GUC* IGS was found to be the main factor driving the size increase of the *A. malesianum* cp genome. This large IGS seems to exist often in fern cp genomes, but unlike previous studies, we noticed that *rpoB-trnD-GUC* IGS affects the dynamic change of IR/SC boundaries in *A. malesianum*. The entrance of the gene into IRs does not cause IR expansion; instead, it destroys the integrity of genes near IRs. This study provides a model to explain how the size increase of *rpoB-trnD-GUC* IGS leads to IR/SC boundaries change, providing a new hypothesis for testing the structural evolution of the cp genome in ferns. However, there are also some limitations. Although we put forward the hypothesis, at present, we have only found this phenomenon in *A. malesianum*. Meanwhile, it is unknown whether the damaged *trnT-UGU* can be expressed. This also suggests that relevant research on the cp genome, especially in IR/SC boundaries analysis, must carefully compare whether the gene is really lost or, as in this study, it is just omitted due to the destruction of its integrity. In conclusion, in future research, we may expand the cp genome of Pteridaceae species and make a detailed comparative analysis to see whether this structural change is universal in this family of ferns. At the same time, if supporting transcriptome data is increased, the research will improve.

## Figures and Tables

**Table 1:** General comparison of six *Adiantum* cp genomes.

Species	LSC		SSC		IR		Size	GC% (Total)
	Length (bp)	GC%	Length (bp)	GC%	Length (bp)	GC%		
*A. flabellulatum*	83,384	42.5	21,449	39.2	23,615	46.7	152,063	43.3
*A. malesianum*	89,030	41.8	21,487	37.7	22,077	46.6	154,671	42.6
*A. capillus-veneris*	82,282	40.8	21,390	37.1	23,448	46.3	150,568	42.0
*A. shastense*	82,113	43.7	21,539	41.6	23,381	46.7	150,414	44.3
*A. reniforme* var. *sinense*	83,267	41.9	21,459	38.1	22,688	46.9	150,102	42.8
*A. nelumboides*	83,281	41.9	21,483	38.1	22,596	46.8	149,956	42.8

**Table 2:** List of primer design and identity to the original sequence.

Name	Location in the cp Genome	Forward Primer	Reverse Primer	Length	Identity
IRa/LSC	153,708–154,676; 1–436	CGGGTTCTTTCCGTTTTCTATTG	TTATACATGAAGGATCCTGTTCTAACATT	1.4 kb	95.0%
LSC/IRb	88,721–89,920	ATCGTTTGAGGAAGTAATTTTTTAATTTTGAC	TTTGTTCCGGACGCCTC	1.2 kb	92.9%
IRb/SSC	110,548–111,947	TACGAATCTCCCCGGATAGG	TTTATTTTCTTACTTTCGAGGGAGATTTTT	1.4 kb	94.9%
SSC/IRa	131,727–133,148	TTACTCTCCTTGCTGTTGGATAATT	TCTCCCCGGATAGGATTCG	1.4 kb	94.8%

## Data Availability

The complete cp genome was deposited in Genbank of the NCBI under the accession numbers NC_064144.1 and NC_063331.

## Supplementary Materials

The following supporting information can be downloaded at: https://www.mdpi.com/article/10.3390/ijms232415616/s1.
