# Supplementary material for: A Large Intergenic Spacer Leads to the Increase in Genome Size and Sequential Gene Movement around IR/SC Boundaries in the Chloroplast Genome of Adiantum malesianum (Pteridaceae)
Source: Int J Mol Sci. 2022 Dec 9;23(24):15616. doi: 10.3390/ijms232415616 (PMC9778900; doi:10.3390/ijms232415616)
Supplement: Supplementary file 1 [file ijms-23-15616-s001.zip › Table S1.pdf]

Table S1 List of genes in the chloroplast genome of *A. flabellulatum* and *A. malesianum*

Category	Gene group	Common gene	Differential gene	
			A. flabellulatum	*A. malesianum*
Photosynthesis	Subunits of photosystem I	*psaA, psaB, psaC, psaI, psaJ*		
	Subunits of photosystem II	*psbA (2), psbB, psbC, psbD, psbE, psbF, psbH, psbI, psbJ, psbK, psbL, psbM, psbN, psbT, psbZ*		
	Subunits of NADH dehydrogenase	*ndhA**, *ndhB**, *ndhC, ndhD, ndhE, ndhF, ndhG, ndhH, ndhI, ndhJ, ndhK*		
	Subunits of cytochrome b/f complex	*petA, petB**, *petD**, *petG, petL, petN*		
	Subunits of ATP synthase	*atpA, atpB, atpE, atpF**, *atpH, atpI*		
	Large subunit of rubisco	*rbcL*		
	Subunits protochlorophyllide reductase	*chlB, chlL, chlN*		
Self-replication	Proteins of large ribosomal subunit	*rpl14, rpl16**, *rpl2**, *rpl20, rpl21, rpl22, rpl23, rpl32, rpl33, rpl36*		
	Proteins of small ribosomal subunit	*rps11,* △*rps12*** *(2), rps14, rps15, rps16**, *rps18, rps19, rps2, rps3, rps4, rps7 (2), rps8*		
	Subunits of RNA polymerase	*rpoA, rpoB, rpoC1**, *rpoC2*		
	Ribosomal RNAs	*rrn16 (2), rrn23 (2), rrn4.5 (2), rrn5 (2)*		
	Transfer RNAs	*trnA-UGC** *(2), trnC-GCA, trnD-GUC, trnE-UUC, trnG-GCC,trnG-UCC**,*trnH-GUG (2) ,trnI-GAU** *(2),trnL-CAA**,*trnL-UAG,trnM-CAU,trnN-GUU (2),trnP-GGG,trnP-UGG,trnQ-UUG,trnR-ACG (2),trnR-UCU,trnS-GCU,trnS-GGA,trnS-UGA,trnT-GGU ,trnV-UAC**,*trnW-CCA,trnY-GUA*	*trnI-CAU, trnT-UGU** *(2), trnF-GAA*	*trnI-CAU (2), trnF-GAA (2)*
Other genes	Maturase	*matK*		
	Protease	*clpP***		
	Envelope membrane protein	*cemA*		
	Acetyl-CoA carboxylase	*accD*		
	c-type cytochrome synthesis gene	*ccsA*		
	Translation initiation factor	*infA*		
Genes of unknown function	Conserved hypothetical chloroplast ORF	*ycf1, ycf2 (2), ycf3***, *ycf4*		*ycf94*

*: Gene with one intron; **: Gene with two introns; (2): Number of copies of multi-copy genes; △: trans-splicing genes.
