# Supplementary material for: A Large Intergenic Spacer Leads to the Increase in Genome Size and Sequential Gene Movement around IR/SC Boundaries in the Chloroplast Genome of Adiantum malesianum (Pteridaceae)
Source: Int J Mol Sci. 2022 Dec 9;23(24):15616. doi: 10.3390/ijms232415616 (PMC9778900; doi:10.3390/ijms232415616)
Supplement: Supplementary file 1 [file ijms-23-15616-s001.zip › Table S2.pdf]

Table S2 List of SSR motifs in six species of *Adiantum*

Motif	*A. flabellulatum*	*A. malesianum*	*A. shastense*	*A. nelumboides*	*A. reniforme* var. *sinense*	*A. capillus-veneris*
A/T	45.83%	61.97%	42.67%	48.15%	52.73%	65.14%
C/G	33.33%	18.31%	26.67%	25.93%	21.82%	21.10%
AG/CT	4.17%	4.23%	2.67%	-	-	1.83%
AT/AT	4.17%	7.04%	8.00%	1.85%	10.91%	1.83%
AC/GT	-	1.41%	5.33%	11.11%	1.82%	-
AAG/CTT	-	1.41%	-	1.85%	1.82%	0.92%
AAT/ATT	-	-	-	-	-	0.92%
AAC/GTT	-	-	2.67%	-	-	-
AGG/CCT	-	2.82%	-	-	-	-
AAAT/ATTT	-	-	-	1.85%	1.82%	-
AAAG/CTTT	-	-	2.67%	-	-	5.50%
AAGG/CCTT	-	-	1.33%	-	-	-
AAGT/ACTT	-	1.41%	-	-	-	-
AATT/AATT	-	1.41%	-	-	-	-
AATC/ATTG	-	-	1.33%	1.85%	1.82%	0.92%
ACAT/ATGT	-	-	-	1.85%	1.82%	-
ACCT/AGGT	-	-	1.33%	-	-	-
ACTC/AGTG	2.08%	-	-	-	-	-
AGAT/ATCT	10.42%	-	4.00%	5.56%	5.45%	1.83%
AAAGT/ACTTT	-	-	1.33%	-	-	-
