# Supplementary material for: A Large Intergenic Spacer Leads to the Increase in Genome Size and Sequential Gene Movement around IR/SC Boundaries in the Chloroplast Genome of Adiantum malesianum (Pteridaceae)
Source: Int J Mol Sci. 2022 Dec 9;23(24):15616. doi: 10.3390/ijms232415616 (PMC9778900; doi:10.3390/ijms232415616)
Supplement: Supplementary file 1 [file ijms-23-15616-s001.zip › Table S3.pdf]

Table S3 List of dispersed repeats information of six species of *Adiantum*

**A. flabellulatum**						
**No.**	**Size**	**Type**	**Start 1**	**Start 2**	**Location**	**Region**
1	88	F	125977	125988	*IGS (ycf1, chlN); IGS (ycf1, chlN)*	SSC; SSC
2	77	F	125977	125999	*IGS (ycf1, chlN); IGS (ycf1, chlN)*	SSC; SSC
3	66	F	125977	126010	*IGS (ycf1, chlN); IGS (ycf1, chlN)*	SSC; SSC
4	60	F	62564	62593	*IGS (petG, trnW-CCA); IGS (petG, trnW-CCA)*	LSC; LSC
5	55	F	125977	126021	*IGS (ycf1, chlN); IGS (ycf1, chlN)*	SSC; SSC
6	61	F	35958	35962	*IGS (trnfM-CAU, rps14); IGS (trnfM-CAU, rps14)*	LSC; LSC
7	51	R	35971	35971	*IGS (trnfM-CAU, rps14); IGS (trnfM-CAU, rps14)*	LSC; LSC
8	49	R	35974	35974	*IGS (trnfM-CAU, rps14); IGS (trnfM-CAU, rps14)*	LSC; LSC
9	48	F	35971	35975	*IGS (trnfM-CAU, rps14); IGS (trnfM-CAU, rps14)*	LSC; LSC
10	47	R	35971	35971	*IGS (trnfM-CAU, rps14); IGS (trnfM-CAU, rps14)*	LSC; LSC
11	45	R	35978	35978	*IGS (trnfM-CAU, rps14); IGS (trnfM-CAU, rps14)*	LSC; LSC
12	44	F	35971	35979	*IGS (trnfM-CAU, rps14); IGS (trnfM-CAU, rps14)*	LSC; LSC
13	44	F	125977	126032	*IGS (ycf1, chlN); IGS (ycf1, chlN)*	SSC; SSC
14	43	R	35971	35971	*IGS (trnfM-CAU, rps14); IGS (trnfM-CAU, rps14)*	LSC; LSC
15	41	R	35982	35982	*IGS (trnfM-CAU, rps14); IGS (trnfM-CAU, rps14)*	LSC; LSC
16	40	F	35971	35983	*IGS (trnfM-CAU, rps14); IGS (trnfM-CAU, rps14)*	LSC; LSC
17	39	R	35971	35971	*IGS (trnfM-CAU, rps14); IGS (trnfM-CAU, rps14)*	LSC; LSC
18	39	R	77954	77954	*IGS (rpl14, rpl16); IGS (rpl14, rpl16)*	LSC; LSC
19	37	R	35986	35986	*IGS (trnfM-CAU, rps14); IGS (trnfM-CAU, rps14)*	LSC; LSC
20	37	R	77954	77954	*IGS (rpl14, rpl16); IGS (rpl14, rpl16)*	LSC; LSC
21	37	F	77954	77956	*IGS (rpl14, rpl16); IGS (rpl14, rpl16)*	LSC; LSC
22	37	R	77956	77956	*IGS (rpl14, rpl16); IGS (rpl14, rpl16)*	LSC; LSC
23	36	F	35971	35987	*IGS (trnfM-CAU, rps14); IGS (trnfM-CAU, rps14)*	LSC; LSC
24	35	R	35971	35971	*IGS (trnfM-CAU, rps14); IGS (trnfM-CAU, rps14)*	LSC; LSC
25	35	R	77954	77954	*IGS (rpl14, rpl16); IGS (rpl14, rpl16)*	LSC; LSC
26	35	F	77954	77958	*IGS (rpl14, rpl16); IGS (rpl14, rpl16)*	LSC; LSC
27	35	R	77958	77958	*IGS (rpl14, rpl16); IGS (rpl14, rpl16)*	LSC; LSC
28	33	R	35990	35990	*IGS (trnfM-CAU, rps14); IGS (trnfM-CAU, rps14)*	LSC; LSC
29	33	R	77954	77954	*IGS (rpl14, rpl16); IGS (rpl14, rpl16)*	LSC; LSC
30	33	F	77954	77960	*IGS (rpl14, rpl16); IGS (rpl14, rpl16)*	LSC; LSC

31	33	R	77960	77960	*IGS (rpl14, rpl16); IGS (rpl14, rpl16)*	LSC; LSC
32	33	F	125977	126043	*IGS (ycf1, chlN); IGS (ycf1, chlN)*	SSC; SSC
33	32	F	35971	35991	*IGS (trnfM-CAU, rps14); IGS (trnfM-CAU, rps14)*	LSC; LSC
34	31	R	35971	35971	*IGS (trnfM-CAU, rps14); IGS (trnfM-CAU, rps14)*	LSC; LSC
35	31	F	62564	62622	*IGS (petG, trnW-CCA); IGS (petG, trnW-CCA)*	LSC; LSC
36	31	R	77954	77954	*IGS (rpl14, rpl16); IGS (rpl14, rpl16)*	LSC; LSC
37	31	F	77954	77962	*IGS (rpl14, rpl16); IGS (rpl14, rpl16)*	LSC; LSC
38	31	R	77962	77962	*IGS (rpl14, rpl16); IGS (rpl14, rpl16)*	LSC; LSC
39	35	R	35967	35967	*IGS (trnfM-CAU, rps14); IGS (trnfM-CAU, rps14)*	LSC; LSC
40	32	F	35967	35991	*IGS (trnfM-CAU, rps14); IGS (trnfM-CAU, rps14)*	LSC; LSC
41	32	R	35991	35994	*IGS (trnfM-CAU, rps14); IGS (trnfM-CAU, rps14)*	LSC; LSC
42	31	R	77952	77954	*IGS (rpl14, rpl16); IGS (rpl14, rpl16)*	LSC; LSC
43	31	F	77952	77962	*IGS (rpl14, rpl16); IGS (rpl14, rpl16)*	LSC; LSC
44	30	R	77963	77964	*IGS (rpl14, rpl16); IGS (rpl14, rpl16)*	LSC; LSC
45	31	R	35967	35967	*IGS (trnfM-CAU, rps14); IGS (trnfM-CAU, rps14)*	LSC; LSC
46	31	F	35967	35995	*IGS (trnfM-CAU, rps14); IGS (trnfM-CAU, rps14)*	LSC; LSC
47	31	R	35995	35995	*IGS (trnfM-CAU, rps14); IGS (trnfM-CAU, rps14)*	LSC; LSC
48	31	R	77952	77952	*IGS (rpl14, rpl16); IGS (rpl14, rpl16)*	LSC; LSC
49	31	R	110948	110948	*IGS (trnP-GGG, trnL-UAG); IGS (trnP-GGG, trnL-UAG)*	SSC; SSC
50	30	P	7654	43557	*trnS-GCU; trnS-GGA*	LSC; LSC
51	30	F	77954	77966	*IGS (rpl14, rpl16); IGS (rpl14, rpl16)*	LSC; LSC
52	30	F	63409	63434	*IGS (trnP-UGG, psaJ); IGS (trnP-UGG, psaJ)*	LSC; LSC
53	30	F	77952	77966	*IGS (rpl14, rpl16); IGS (rpl14, rpl16)*	LSC; LSC

A. malesianum

No.	Size	Type	Start 1	Start 2	Location	Region
1	123	P	52644	68274	*trnF-GAA (partial: 59.35%); trnF-GAA (partial: 59.35%)*	LSC; LSC
2	40	P	31010	32621	*IGS (rpoB, trnD-GUC); IGS (rpoB, trnD-GUC)*	LSC; LSC
3	42	F	30684	30731	*IGS (rpoB, trnD-GUC); IGS (rpoB, trnD-GUC)*	LSC; LSC
4	36	F	32524	32626	*IGS (rpoB, trnD-GUC); IGS (rpoB, trnD-GUC)*	LSC; LSC
5	35	P	31010	32524	*IGS (rpoB, trnD-GUC); IGS (rpoB, trnD-GUC)*	LSC; LSC
6	39	F	30631	30649	*IGS (rpoB, trnD-GUC); IGS (rpoB, trnD-GUC)*	LSC; LSC
7	36	P	41533	41533	*IGS (psbD, trnT-GGU); IGS (psbD, trnT-GGU)*	LSC; LSC
8	37	P	42199	42199	*IGS (trnfM-CAU, rps14); IGS (trnfM-CAU, rps14)*	LSC; LSC

9	33	P	30913	32614	*IGS (rpoB, trnD-GUC); IGS (rpoB, trnD-GUC)*	LSC; LSC
10	32	P	41533	41539	*IGS (psbD, trnT-GGU); IGS (psbD, trnT-GGU)*	LSC; LSC
11	31	F	74424	84313	*clpP-intron1; IGS (rpl14, rpl16)*	LSC; LSC
12	30	P	8565	49837	*trnS-GCU; trnS-GGA*	LSC; LSC
13	30	F	30913	31024	*IGS (rpoB, trnD-GUC); IGS (rpoB, trnD-GUC)*	LSC; LSC

A. capillus-veneris

No.	Size	Type	Start 1	Start 2	Location	Region
1	36	P	35394	35394	*IGS (trnfM, rps14); IGS (trnfM, rps14)*	LSC; LSC
2	32	P	76784	76820	*IGS (rpl14, rpl16); IGS (rpl14, rpl16)*	LSC; LSC
3	32	P	34648	34648	*IGS (psbD, trnT); IGS (psbD, trnT)*	LSC; LSC
4	30	P	7212	42947	*trnS; trnS-3*	LSC; LSC
5	30	P	69752	69787	*IGS (psbT, psbN); psbN (partial: 40.00%)*	LSC; LSC

A. shastense

No.	Size	Type	Start 1	Start 2	Location	Region
1	66	P	109436	109436	*IGS (trnP, trnL-TAG); IGS (trnP, trnL-TAG)*	SSC; SSC
2	34	P	76820	76820	*IGS (rpl14, rpl16); IGS (rpl14, rpl16)*	LSC; LSC
3	30	R	48517	48519	*trnV-TAC-intron; trnV-TAC-intron*	LSC; LSC
4	32	R	13061	13061	*IGS (atpF, atpH); IGS (atpF, atpH)*	LSC; LSC
5	31	R	48518	48518	*trnV-TAC-intron1; trnV-TAC-intron1*	LSC; LSC
6	30	P	31311	31311	*IGS (trnS-TGA, psbC); IGS (trnS-TGA, psbC)*	LSC; LSC
7	30	R	48511	63798	*trnV-TAC-intron; IGS (rps18, rpl20)*	LSC; LSC
8	30	F	48517	48518	*trnV-TAC-intron; trnV-TAC-intron1*	LSC; LSC
9	30	F	63790	63791	*IGS (rps18, rpl20); IGS (rps18, rpl20)*	LSC; LSC
10	30	C	116406	116407	*nadA-intron; nadA-intron*	SSC; SSC
11	30	P	116411	116411	*nadA-intron; nadA-intron*	SSC; SSC
12	32	F	60050	60053	*IGS (petA, psbJ); IGS (petA, psbJ)*	LSC; LSC
13	31	P	3079	3113	*IGS (matK, ycf94); IGS (matK, ycf94)*	LSC; LSC
14	31	R	29817	60050	*IGS (petN, trnC-GCA); IGS (petA, psbJ)*	LSC; LSC
15	31	R	29819	85256	*trnC-GCA (partial: 3.23%); rrn5 (partial: 19.35%)*	LSC; IRb
16	31	C	29819	147244	*trnC-GCA (partial: 3.23%); rrn5-2 (partial: 12.90%)*	LSC; IRa
17	31	P	35226	35260	*IGS (trnT-GGT, trnfM-CAT); IGS (trnT-GGT, trnfM-CAT)*	LSC; LSC
18	31	F	48514	55125	*trnV-TAC-intron; IGS (accD, psaI)*	LSC; LSC
19	30	P	7917	31161	*trnS-GCT; trnS-TGA (partial: 96.67%)*	LSC; LSC

20	30	C	48517	116078	*trnV-TAC-intron; nadA-intron*	LSC; SSC
21	30	C	48517	116086	*trnV-TAC-intron; nadA-intron*	LSC; SSC
22	30	R	55127	63795	*IGS (accD, psaI); IGS (rps18, rpl20)*	LSC; LSC
23	30	R	60047	63793	*IGS (petA, psbJ); IGS (rps18, rpl20)*	LSC; LSC
24	30	F	63790	63792	*IGS (rps18, rpl20); IGS (rps18, rpl20)*	LSC; LSC
25	30	F	63793	85255	*IGS (rps18, rpl20); rrn5 (partial: 13.33%)*	LSC; IRb
26	30	P	63793	147246	*IGS (rps18, rpl20); rrn5-2 (partial: 6.67%)*	LSC; IRa

A. nelumboides

No.	Size	Type	Start 1	Start 2	Location	Region
1	120	P	46166	61856	*trnF-GAA (partial: 60.83%); trnF-GAA (partial: 60.83%)*	LSC; LSC
2	58	P	66620	66620	*IGS (rps12, clpP); IGS (rps12, clpP)*	LSC; LSC
3	34	P	35854	35854	*IGS (trnfM-CAU, rps14); IGS (trnfM-CAU, rps14)*	LSC; LSC
4	32	P	10422	10422	*IGS (trnG-UCC, trnR-UCU); IGS (trnG-UCC, trnR-UCU)*	LSC; LSC
5	34	F	36630	38845	*psaB; psaA*	LSC; LSC
6	31	F	35855	109803	*IGS (trnfM-CAU, rps14); IGS (trnP-GGG, trnL-UAG)*	LSC; SSC
7	31	P	35856	109803	*IGS (trnfM-CAU, rps14); IGS (trnP-GGG, trnL-UAG)*	LSC; SSC

A. reniforme* var. *sinense

No.	Size	Type	Start 1	Start 2	Location	Region
1	120	P	46153	61843	*trnF-GAA (partial: 60.83%); trnF-GAA (partial: 60.83%)*	LSC; LSC
2	58	P	66608	66608	*clpP-intron; clpP-intron*	LSC; LSC
3	34	P	35841	35841	*IGS (trnfM-CAU, rps14); IGS (trnfM-CAU, rps14)*	LSC; LSC
4	32	P	10421	10421	*IGS (trnG-UCC, trnR-UCU); IGS (trnG-UCC, trnR-UCU)*	LSC; LSC
5	34	F	36617	38832	*psaB; psaA*	LSC; LSC
6	31	F	35842	109882	*IGS (trnfM-CAU, rps14); IGS (trnP-GGG, trnL-UAG)*	LSC; SSC
7	31	P	35843	109882	*IGS (trnfM-CAU, rps14); IGS (trnP-GGG, trnL-UAG)*	LSC; SSC
