# Supplementary material for: A Large Intergenic Spacer Leads to the Increase in Genome Size and Sequential Gene Movement around IR/SC Boundaries in the Chloroplast Genome of Adiantum malesianum (Pteridaceae)
Source: Int J Mol Sci. 2022 Dec 9;23(24):15616. doi: 10.3390/ijms232415616 (PMC9778900; doi:10.3390/ijms232415616)
Supplement: Supplementary file 1 [file ijms-23-15616-s001.zip › Table S4.pdf]

Table S4 Size and distribution of tandem repeats in the chloroplast genome of *Adiantum*

Species	Motif	Copy number	Start-End	Location
A. flabellulatum	22	2	20648-20691	*IGS (rpoC2, rpoC1); partial rpoC1 (24 bp)*
	24	2	34728-34775	*IGS (psbD, trnT-GGT)*
	15	2.1	44803-44833	*IGS (rps4, trnL-CAA)*
	29	3.1	62565-62653	*IGS (petG, trnW-CCA)*
	25	2	63410-63459	*IGS (trnP-UGG, psaJ)*
	15	2.1	90024-90055	*IGS (rrn23, trnA-UGC)*
	18	2.1	90045-90081	*IGS (rrn23, trnA-UGC)*
	20	2	97062-97102	*IGS (rps7, psbA)*
	20	2	120052-120092	*IGS (ndhH, rps15)*
	20	2	138346-138386	*IGS (psbA, rps7)*
	18	2.1	145367-145403	*IGS (trnA-UGC, rrn23)*
	15	2.1	145393-145424	*IGS (trnA-UGC, rrn23)*
A. malesianum	102	1.9	32506--32695	*IGS (rpoB, trnD-GUC)*
	47	3.7	30615--30772	*IGS (rpoB, trnD-GUC)*
	27	1.9	30770--30821	*IGS (rpoB, trnD-GUC)*
	22	7.5	30613--30772	*IGS (rpoB, trnD-GUC)*
	20	2	84016--84054	*IGS (rpl14, rpl16)*
	19	2.1	71470--71508	*IGS (rpl20, rps12)*
	19	2.1	103113--103152	*IGS (psbA, trnH-GUG)*
	19	2.1	140550--140589	*IGS (trnH-GUG, psbA)*
	18	4.4	30640--30726	*IGS (rpoB, trnD-GUC)*
	17	2	14467--14500	*IGS (atpH, atpI)*
	16	2.1	115089--115121	*IGS (trnP-GGG, trnL-UAG)*
	15	2.1	80666--80696	*IGS (petD, rpoA)*
A. capillus-veneris	15	2.1	76199-76229	*IGS (rpsB, rpl14)*
A. shastense	18	2	26536-26571	*IGS (rpoB, trnD-GTC)*
	24	2	34215-34262	*IGS (psbD, trnT-GGT)*
	23	2	113486-113531	*IGS (psaC, ndhE), partial ndhE (7 bp)*
